# Remedial Effect of Intravenous Cyclophosphamide in Corticosteroid-Refractory Patients in the Acute Phase of Neuromyelitis Optica Spectrum Disorder-Related Optic Neuritis

**DOI:** 10.3389/fneur.2020.612097

**Published:** 2021-01-15

**Authors:** Ling Wang, Kaiqun Liu, Xiao Tan, Lin Zhou, Yuxin Zhang, Xiaoning Liu, Yue Fu, Wei Qiu, Hui Yang

**Affiliations:** ^1^State Key Laboratory of Ophthalmology, Zhongshan Ophthalmic Center, Sun Yat-sen University, Guangzhou, China; ^2^Department of Neurology, Third Affiliated Hospital of Sun Yat-sen University, Guangzhou, China

**Keywords:** neuromyelitis optica spectrum disorder, optic neuritis, cyclophosphamide, methylprednisolone, effect

## Abstract

**Background:** To investigate the remedial efficacy and safety of intravenous cyclophosphamide (CP) in the acute phase in patients with neuromyelitis optica spectrum disorder-related optic neuritis (NMOSD-ON) who are refractory to intravenous methylprednisolone (MP) treatment.

**Design:** This study was a single-center, retrospective, observational case-control cohort study.

**Methods:** Thirty-six patients who had acute NMOSD-ON attacks and were refractory to MP treatment were included. Patents were divided into two groups: the remedial CP group, and the MP group. The best-corrected visual acuity (BCVA), mean deviation (MD) of the visual field (VF), visual evoked potential amplitude (VEP-A), visual evoked potential latency (VEP-T), and average thickness of the retinal nerve fiber layer (RNFL) at onset, 1 month (m), 3 m, and 6 m after the attack were analyzed. Routine blood test results, liver and kidney function, routine urinalysis results and general condition were analyzed for safety issues at each follow-up. Fisher's exact test, the Mann-Whitney U test, the Kruskal-Wallis test and the Wilcoxon rank-sum test were used for statistical analysis.

**Results:** The remedial CP group showed significant improvement over 6 m with regard to BCVA and MD (*P* < 0.05),whereas MP group only showed significant improvement in MD (*P* < 0.05). Regarding remedial CP intervention time window, the CP ≤ 30 days group showed significant improvement over 6 m with regard to BCVA (*P* = 0.002), MD (*P* = 0.003), and VEP-A (*P* = 0.036), while those CP > 30 days group did not. Both two subgroups showed significantly RNFL thickness reduction, however, BCVA, MD, VEP-A, VEP-T, and RNFL thickness showed no significant differences between the two subgroups at any follow-up point (*P* > 0.05).

**Conclusion:** CP within 30 days of attack onset is safe and might have a beneficial degree of therapeutic efficacy for acute-phase treatment of NMOSD-ON that is refractory to MP treatment alone.

## Introduction

Neuromyelitis optica spectrum disorders (NMOSD), which include the neuromyelitis optica (NMO), previously known as Devic's syndrome, are defined as a group of inflammatory disorders of the central nervous system(CNS) characterized by episodes of immune-mediated demyelination and axonal damage preferentially affecting the optic nerves and spinal cord (1); these disorders are mainly associated with aquaporin-4 [AQP4–immunoglobulin G (IgG)] seropositivity.

The visual outcome of NMOSD-ON is often devastating, with one or two acute attacks potentially leading to irreversible blindness. The treatment regimen includes rescue in the acute phase and effective prevention of relapses later on. Treatment in the acute phase is of utmost importance and requires measures that work quickly. At present, the available clinical choices include intravenous methylprednisolone (MP), plasma exchange (PE) or immunoadsorption (IA), cyclophosphamide (CP), intravenous immunoglobulin (IVIg), rituximab and eculizumab (2). Among these treatments, intravenous CP has the advantages of speed, low cost, and easily obtained materials. It has been widely used for many years to treat a variety of autoimmune diseases, such as neuropsychiatric systemic lupus erythematosus (NPSLE) (3) and Sjögren's syndrome (SS) (4).

Based on small case series and retrospective cohort studies of the effect of CP on NMOSD, the European Federation of Neurological Societies (EFNS) recommends CP treatment as second-line therapy for patients with NMO, especially in cases associated with SLE (5). Several studies (6, 7) of CP alone or in combination with other therapies have shown an effect on NMOSD-related longitudinally extensive transverse myelitis (NMOSD-LETM). However, to the best of our knowledge, no observations have been made on the remedial effect of CP on NMOSD-ON, which is a superior model for observing the effect of CP on functional and structural changes in a single nerve.

Therefore, the aim of this study was to thoroughly evaluate the remedial effect and safety of intravenous CP at the acute phase of NMOSD-ON by analyzing the best-corrected visual acuity (BCVA), visual field (VF), visual evoked potentials (VEPs), optical coherence tomography (OCT) results and other safety indices at each follow-up.

## Materials and Methods

### Patients and Design

This single-center, retrospective, observational case-control cohort study was approved by the Ethics Committee of Zhongshan Ophthalmology Center of Sun Yat-sen University in 2014 (Ethics No. 2014 meky049).

In 91 NMOSD-ON patients admitted at Zhongshan Ophthalmic Center, Sun Yat-sen University, between June 2013 and March 2020 following the clinical onset of an acute attack, the following clinical information was retrospectively reviewed: serum AQP4 antibody status, ophthalmology and general medical history, comprehensive ophthalmic examination data, immunological examination and craniocerebral magnetic resonance imaging (MRI) examination, treatment regimen, and effects of treatment at each follow-up.

### Subjects

The eligibility criteria were as follows: (1) AQP4-IgG–positive optic neuritis patients, meeting the NMOSD 2015 diagnostic criteria (8); (2) having no relapse at least 90 days prior to the acute attack; (3)age ≥ 18 years or older; (4) ≥6 m regular follow-up after onset, with complete records of BCVA, VF, VEP, and OCT at each follow-up; (5) records of liver and kidney function tests and routine blood tests at baseline and each follow-up; (6) unresponsive to an MP regimen. Treatment response was assessed at 14 days after the initiation of MP treatment. MP nonresponders were defined as (1) having visual acuity improvement <2 lines of the Snellen chart with a baseline visual acuity ≥ 0.1 (9); (2) having improvement <0.1 with a baseline visual acuity of ~0.02 (counting fingers, or CF); (3) if the baseline visual acuity was worse than CF, such as no light perception (NLP), light perception (LP) or hand motion (HM), improvement from NLP to LP, from LP to HM, or from HM to CF was considered a response to MP treatment. If there was no treatment response, patients were considered to be refractory to MP treatment.

The exclusion criteria were as follows: (1) received remedial treatment other than MP and CP for NMOSD-ON; (2) responded well to MP; (3) conditions that render the side effects of CP unacceptable, for example, fertility requirements, prior blood disorders such as anemia, liver dysfunction, and kidney dysfunction.

### Group Division

**Figure 1:**
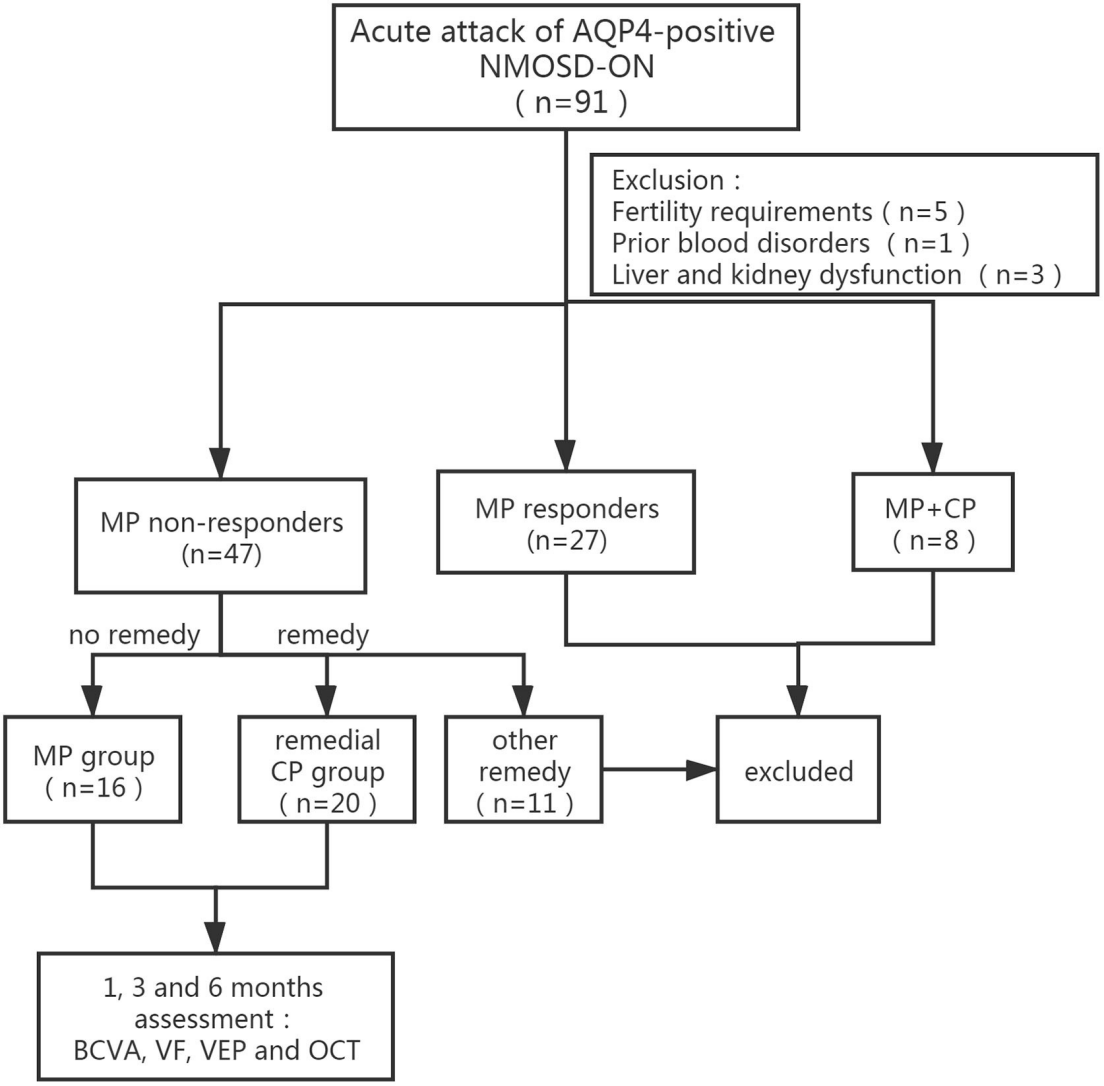
Flow diagram of the patients group division according to MP response and subsequent remedical strategy. NMOSD-ON, neuromyelitis optica spectrum disorders related optic neuritis; AQP-4, aquaporin-4; MP, intravenous methylprednisolone; CP, intravenous cyclophosphamide; BCVA, best-corrected visual acuity; VF, visual field; VEP, visual evoked potential.

Among 91 acute NMOSD-ON patients, 27 patients who responded well to initial MP therapy were excluded from the study. Among the remaining patients, 11 patients who received remedial treatment other than MP and CP, such as PE (2 patients), IA (1 patient), rituximab (1 patient), and IVIg (7 patients), were excluded. Five patients had fertility requirements, 1 patient had prior blood disorders, and 3 patients had liver and kidney dysfunction. Eight patients who had received MP and CP therapy simultaneously from very beginning were excluded. Twenty patients who received remedial CP therapy were defined as the remedial CP group, and 16 patients who received no other remedial therapy were defined as the MP group (Table 1 and Figure 1).

**Table 1:** Basic characteristics of AQP4-positive NMOSD-ON patients in each group.

	**Remedial CP group (*n* = 20)**	**MP group (*n* = 16)**	***P* value**
Age at onset, year, median (IQR)	41.00 (33.50, 51.00)	37.00 (31.00, 40.00)	0.24
Female, *n* (%)	19 (95%)	14 (87.5%)	0.57
Previous attacks, *n*, median (IQR)	2 (2, 2)	2.5 (1, 4)	0.39
Recurrent attack, *n* (%)	16 (80%)	11 (68.75%)	0.47
Unilateral involvement, *n* (%)	17 (85%)	15 (93.75%)	0.61
Merge multifocal involvement			
Brain, *n* (%)	1 (5%)	0	–
Spinal cord, *n* (%)	0	0	–
Abnormal HLA-B27, *n* (%)	1 (5%)	1 (6.25%)	>0.99
Abnormal autoantibodies			
ANA + SSA + SSB, *n* (%)	8 (40%)	4 (25%)	>0.99
TPOAb + TGAb, *n* (%)	2(10%)	0	–
Disease duration, year, median (IQR)	4 (2.25, 5)	5.5 (1.5, 9.75)	0.35
Time from onset to MP, days; median (IQR)	9 (2, 11.75)	7 (4, 14.25)	0.86
Time from onset to CP, days; median (IQR)	26 (13.5, 44)	–	–
Pretreatment, *n* (%)			
Oral CS	6 (30%)	4 (25%)	>0.99
Immunosuppressants	4 (20%)	3 (18.75%)	>0.99
IVMP	2 (10%)	1 (6.25%)	>0.99
IVIG	0	0	–
Plasmapheresis	0	0	–
Follow-up, month, median (IQR)	22.00 (11.25, 33.00)	23.00 (10.50, 31.75)	0.82
Annual relapse rate, median (IQR)	0.5 (0.5, 2)	1.5 (1, 2)	0.26
Recurrent cases, *n*	3	4	0.68
Baseline BCVA, LogMar, median (IQR)	1.52 (0.8, 1.96)	1.62 (0.38, 2)	0.89
Baseline MD, dB, median (IQR)	−34.00 (−34.00, −21.45)	−34.00 (−34.00, −18.52)	0.58
Baseline VEP-A, μV, median (IQR)	4.24 (2.13, 6.05)	0.00 (0.00, 4.08)	0.07
Baseline abnormal VEP-T, *n* (%)	10/15 (66.7%)	10/15 (66.7%)	>0.99
Baseline RNFL, μm, median (IQR)	106.50 (73.25, 129.75)	90.50 (66.25, 105.75)	0.12

MP, intravenous methylprednisolone; CP, intravenous cyclophosphamide; CS, corticosteroid; BCVA, best-corrected visual acuity; MD, mean deviation; VEP-A, visual evoked potential amplitude; VEP-T, visual evoked potential latency; RNFL, retinal nerve fiber layer.

### Treatment Regimen

In the MP group, a pulse MP regimen was given intravenously: 1 g/day × 3–5 days, 0.5 g/day × 3 days, 0.25 g/day × 3 days, 0.12–0.125 g/day × 3 days, followed by oral prednisone tablets at 1–2 mg/kg body weight per day, gradually tapering off for no less than 6 m. In the remedial CP group, the MP pulse regimen was the same as that in the MP group, and a CP of 375 mg/mm^2^ (0.6 g) weekly over various periods (1–4 w) was given as remedial treatment 2 weeks after initial MP pulse therapy. In all groups, mycophenolate mofetil (MMF) 2 mg/kg/day was given 1 month after onset to prevent relapse.

### Clinical Assessment

#### Ophthalmic Evaluation

(1) BCVA was recorded using Snellen charts and transformed into the logarithm of the minimum angle of resolution (logMAR) using the formula logMAR = –logBCVA. FC, HM, LP, and NLP were converted to 1.85, 2.0, 2.7, and 3.0, respectively (10). (2)The VF was assessed using the Humphrey Field Analyzer (STATPAC, Allergan Humphrey, San Leandro, Calif), and the MD of the central 30-2 SITA program was used to record visual field defects. It was required that false negative rate or false positive rate <15%, fixation loss <30%, Otherwise, VF could be repeated until the quality had met the standard. (3)Visual evoked potentials (VEPs, RETI-Port/Scan 21, Germany): VEP amplitude (VEP-A) and VEP latency (VEP-T) were recorded for a stimulus size of 60 min of arc. When no signal was recorded due to poor vision, VEP-A was recorded as 0 μV. VEP-T was considered as abnormal when longer than 112 ms or when no clear signal could be evoked due to poor vision (11). (4) OCT was performed using HD-OCT (Heidelberg Engineering, Heidelberg, Germany). The average thickness of the retinal nerve fiber layer (RNFL) was analyzed and defined as RNFL thickness in the diameter of 3.4 mm around the optic disc. BCVA, VF, VEP, and OCT recordings in all patients at baseline, 1, 3, and 6 m were analyzed.

#### Immune Examination at Baseline

(1) Thyroid function and anti-thyroglobulin DNA, anti-thyroglobulin antibody, anti-thyroid peroxidase antibody; (2) anti-nuclear antibody (anti-dsDNA antibody, anti-nucleosome antibody, anti-histone antibody, anti-U1RNP antibody, anti-SM antibody, anti-SSA antibody, anti-SSB antibody), anti-HLA B27 antibody, anticardiolipin antibody, and rheumatoid factor were analyzed to understand the immune status of the patients and the condition of systemic immune diseases.

#### MRI (Plain Scan + Enhanced)

Baseline brain MRI of each patient was analyzed to exclude other diseases, such as tumors, and the involvement of the brain was analyzed as well.

#### Safety Index

Liver and kidney function and routine blood tests were performed at each follow-up and analyzed for any adverse effects.

The clinical parameters, such as BCVA, MD of VF, VEP-A, VEP-T, and RNFL thickness, and the changes in all these indices (Δ) at each follow-up time point with respect to baseline, i.e., 0–1 m, 0–3 m, and 0–6 m, were compared within each group and between different groups.

The primary outcomes are improvement of visual function (BCVA, VF, VEP) and thinning of the RNFL. The secondary outcomes are the safety indexes.

### Statistical Analysis

Statistical analyses were carried out using SPSS Statistics version 19 (SPSS Inc., Chicago, IL, USA). The figures were constructed using GraphPad Prism, version 8 (GraphPad Software, La Jolla, CA). The data of affected eyes were included in unilateral ON cases, and data of one randomly chosen eye were included when both eyes were affected simultaneously. Continuous variables were presented as medians with interquartile ranges (IQRs). The Mann-Whitney U test, Kruskal-Wallis test and Wilcoxon rank-sum test were applied for comparison as appropriate. Categorical variables were compared using the chi-square test and Fisher's exact test. A 2-sided *P* value < 0.05 was considered to be statistically significant. A binary logistic regression was used to analyze weather time to CP intervention represents an important influencing factors. Data was reported with odds ratio (OR) and 95% confidence intervals (CI).

## Results

### Baseline Characteristics (Table 1)

All 36 patients with acute NMOSD-ON were included: 20 patients in the remedial CP group, and 16 patients in the MP group. Regarding the baseline clinical characteristics, including age, sex ratio, recurrent state of the attack, follow-up period, brain or spinal cord involvement and immune disease status, no significant differences were found between the two groups. Regarding the ophthalmic characteristics, including BCVA, MD of VF, VEP-A and VEP-T, no significant differences were found between the two groups.

### Clinical Outcome

#### Comparison Between the Remedial CP Group and the MP Group (Table 2)

**Figure 2:**
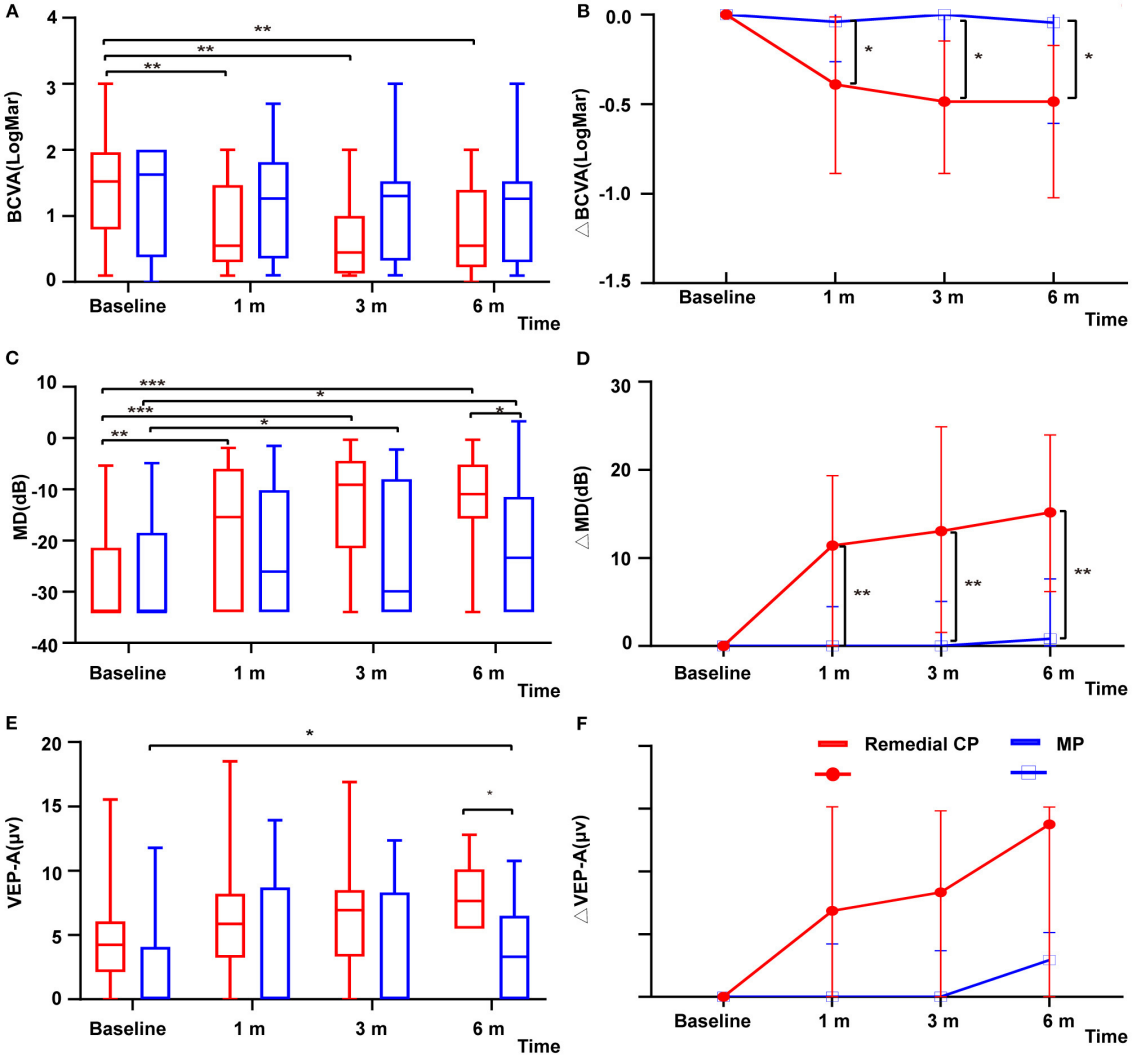
**(A)** Distribution of BCVA among patients with remedial CP therapy and MP therapy over the course of follow-up. The red boxes represent the group that received MP alone. Box plot details: thick horizontal bar: median; box: interquartile range (25–75%). The *P* values for the difference between the BCVA values of the remedial CP group and the MP group were 0.89, 0.15, 0.12, and 0.32 at baseline, 1, 3, and 6 m of follow-up, respectively. **(B)** Comparison of changes in ΔBCVA among patients with remedial CP therapy and MP therapy: The *P* values for the difference between the ΔBCVA values of the remedial CP group and the MP group were 0.02, 0.01, and 0.02 at 1, 3, and 6 m of follow-up, respectively. **(C)** Distribution of MD among patients with remedial CP therapy and MP therapy. The red boxes represent the group that received MP alone. Box plot details: thick horizontal bar: median; box: interquartile range (25–75%). The *P* values for the difference between the MD values of the remedial CP group and MP group were 0.58, 0.26, 0.07, and 0.03 at baseline, 1, 3, and 6 m of follow-up, respectively. **(D)** Comparison of changes in ΔMD among patients with remedial CP therapy and MP therapy: The *P* values for the difference between the ΔMD values of the remedial CP group and the MP group were 0.005, 0.003 and 0.003 at 1, 3, and 6 m of follow-up, respectively. **(E)** Distribution of VEP-A among patients with remedial CP therapy and MP therapy: The red boxes represent the group that received MP alone. Box plot details: thick horizontal bar: median; box: interquartile range (25–75%). The *P* values for the difference between the VEP-A values of the remedial CP group and the MP group were 0.07, 0.11, 0.14, and 0.02 at baseline, 1, 3, and 6 m of follow-up, respectively. **(F)** Comparison of changes in ΔVEP-A among patients with remedial CP therapy and MP therapy: The *P* values for the difference between the ΔVEP-A values of the remedial CP group and the MP group were 0.49, 0.33 and 0.17 at 1, 3, and 6 m of follow-up, respectively. *P < 0.05, **P < 0.01, ***P < 0.001.

The remedial CP group showed significant improvement in BCVA as early as 1 m after onset. Significant differences were found between 1 m and baseline (*P* = 0.002). No significant difference was found after 1 m, which might indicate that the BCVA improved rapidly after remedial CP treatment and then leveled off. However, in the MP group, no significant difference was found at any follow-up point compared to baseline (Figure 2A). This difference in BCVA improvement between the two groups was more obvious if the BCVA change to baseline was compared (Figure 2B). The remedial CP group showed significantly better ΔBCVA improvement than the MP group at every follow-up time point (*P* Δ1 m = 0.02, *P* Δ3 m = 0.01, *P* Δ6 m = 0.02).

**Table 2:** Visual function and RNFL in the acute phase of NMOSD-ON: remedial CP group vs. MP group.

**parameter**	**Remedial CP group**			**MP group**			
	**Baseline**	**6 m**	***P*^**a**^**	**Baseline**	**6 m**	***P*^**b**^**	***P*^**c**^**
BCVA (logMAR), median (IQR)	1.52 (0.8, 1.96)	0.55 (0.23, 1.39)	0.001^*^	1.62 (0.38, 2)	1.26 (0.30, 1.52)	0.17	0.32
MD (dB), median (IQR)	−34.00 (−34.00, −21.45)	−10.96 (−15.72, −5.18)	0.000^*^	−34.00 (−34.00, −18.52)	−23.38 (−34.00, −11.52)	0.037^*^	0.03^*^
VEP-A (μv), median (IQR)	4.24 (2.13, 6.05)	7.64 (5.50, 10.10)	0.096	0.00 (0.00, 4.08)	3.31 (0.00, 6.49)	0.014^*^	0.02^*^
Abnormal VEP-T, *n* (%)	10/15 (66.7%)	10/15 (66.7%)	>0.99	10/15 (66.7%)	12/15 (80.00%)	0.68	0.68
RNFL (μm), median (IQR)	106.50 (73.25, 129.75)	51.00 (39.75, 67.50)	0.000^*^	90.50 (66.25, 105.75)	56.50 (45.75, 77.00)	0.000^*^	0.26

P^a^-value for outcomes comparison between baseline and 6 m in the remedial CP group.

P^b^-value for outcomes comparison between baseline and 6 m in the MP group.

P^c^-value for final outcome comparison between the remedial CP group and the MP group.

* P < 0.05.

CP, intravenous cyclophosphamide; MP, intravenous methylprednisolone; BCVA, best-corrected visual acuity; MD, mean deviation; VEP-A, visual evoked potential amplitude; VEP-T, visual evoked potential latency; RNFL, retinal nerve fiber layer; IQR, interquartile range.

Within the remedial CP group, significant improvement in MD was shown as early as 1 m after onset. Significant differences were found between 1 m and baseline (*P* = 0.001), 3 m and 1 m (*P* = 0.039), and no significant differences were found between 6 and 3 m (*P* = 0.88). The MP group also demonstrated significant improvement at 3 m compared to baseline (*P* = 0.025), but no significant difference was found afterward (Figure 2C). Comparison between the two groups: At every follow-up point, the remedial CP group showed better MD improvement than the MP group. However, a significant difference was found only at 6 m (*P* = 0.03). Regarding MD change to baseline, significantly better improvement was found in the remedial CP group compared to the MP group (*P* Δ1 m = 0.005, *P* Δ3 m = 0.003, *P* Δ6 m = 0.003) (Figure 2D).

VEP data were available for analysis in 30 patients, comprising 15 patients in the MP group and 15 in the remedial CP group. Regarding VEP-A, within the remedial CP group, no significant difference could be found at any follow-up point compared to baseline. Within the MP group, significant improvement at 6 m was found compared to baseline (*P* = 0.014) (Figure 2E). Significantly better improvement at 6 m was found in the remedial CP group compared to the MP group (*P* = 0.02). Regarding VEP-A change to baseline, no significant difference was found between the remedial CP group and MP group (*P* Δ1 m = 0.49, *P* Δ3 m = 0.33, *P* Δ6 m = 0.17) (Figure 2F). Regarding the proportion of abnormal VEP-T, no significant difference could be found between the two groups at any follow-up point (*P* > 0.05).

**Figure 3:**
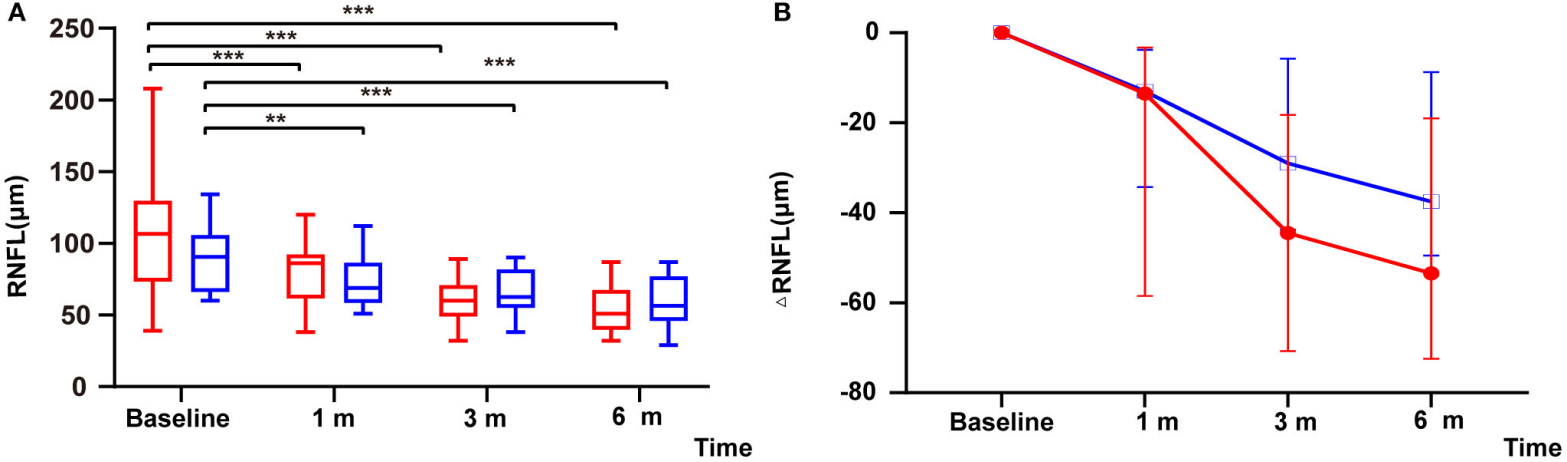
**(A)** Distribution of RNFL among patients with remedial CP therapy and MP therapy: The red boxes represent the group that received MP alone. Box plot details: thick horizontal bar: median; box: interquartile range (25–75%). The *P* values for the difference between the RNFL thicknesses of the remedial CP group and the MP group were 0.12, 0.34, 0.54, and 0.26 at baseline, 1, 3, and 6 m of follow-up, respectively. **(B)** Comparison of changes in ΔRNFL among patients with remedial CP therapy and MP therapy: The *P* values for the difference between the ΔRNFL values of the remedial CP group and the MP group were 0.60, 0.07 and 0.06 at 1, 3, and 6 m of follow-up, respectively. *P < 0.05, **P < 0.01, ***P < 0.001.

Within the remedial CP group, significant thinning in RNFL was found as early as 1 m after onset and continued to 6 m after onset: significant differences were found between 1 m and baseline (*P* = 0.000), 3 and 1 m (*P* = 0.000), and 3 and 6 m (*P* = 0.000). Within the MP group, similar to the remedial CP group, significant thinning was found as early as 1 m after onset and continued to 6 m after onset: significant differences were found between 1 m and baseline (*P* = 0.001), 3 and 1 m (*P* = 0.002), 3 and 6 m (*P* = 0.001). However, no significant difference could be found between the two groups at any follow-up point (Figure 3A). Regarding RNFL change to baseline: No significant difference was found between the two groups at any follow-up point (*P* Δ1 m = 0.60, *P* Δ3 m = 0.07, *P* Δ6 m = 0.06) (Figure 3B).

#### Remedial CP Interference Time Point (Table 3)

CP intervention timepoint was negatively associated with BCVA improvement (OR = 0.89, 95% CI 0.82–1.00, *p* = 0.03). The remedial CP group was further divided into two groups according to the intervention time point of CP: the CP ≤ 30 days group and the CP > 30 days group. Overall, 60% of patients (12/20) received remedial CP treatment within 30 days of onset, while 40% (8/20) of patients received remedial CP treatment after 30 days.

**Table 3:** Clinical outcomes for remedial CP Groups:CP interference time point.

	**Remedial CP ≤ 30 days (*n* = 12)**	**Remedial CP > 30 days (*n* = 8)**	***P* value**
Age year (median, IQR)	41.00 (34.25, 51.00)	39.50 (29.00, 53.75)	0.85
Sex, female/male, *n* (%)	12/0	7/1 (700.00%)	0.40
Recurrent attack, *n* (%)	10 (83.30%)	6 (75.00%)	1.00
**Abnormal autoantibodies**			
HLA B27, *n* (%)	1 (8.30%)	0	0.23
ANA + SSA + SSB, *n* (%)	6 (50%)	2 (25.00%)	
TPOAb + TGAb, *n* (%)	0 (0%)	2 (25.00%)	
Time from onset to MP, days (median, IQR)	9.00 (3.00, 10.75)	6.00 (0.50, 21.25)	0.97
Time from onset to CP, days (median, IQR)	16.00 (12.25, 24.25)	45.50 (37.25, 62.25)	0.00^*^
CP dose totally, median (IQR)	1.2 (0.6, 1.2)	0.9 (0.6, 1.2)	0.85
**BCVA, logMAR, median (IQR)**			
Onset	1.61 (1.11, 1.96)	0.95 (0.15, 1.88)	0.18
1 m	0.55 (0.30, 1.23)	0.66 (0.12, 1.65)	0.97
3 m	0.44 (0.24, 0.92)	0.65 (0.10, 1.65)	0.79
6 m	0.55 (0.30, 1.00)	0.65 (0.12, 1.65)	0.97
ΔBCVA (0–1 m)	−0.67 (−1.08, −1.83)	−0.1 (−0.44, −0.08)	0.02^*^
ΔBCVA (0–3 m)	−0.84 (−1.35, −0.33)	−0.25 (−0.25, 0)	0.01^*^
ΔBCVA (0–6 m)	−0.93 (−1.25, −0.33)	−0.30 (−0.49, 0.08)	0.02^*^
*P* value (onset vs. 6 m)	0.002^*^	0.31	–
**MD, dB, median(IQR)**			
Onset	−34.00 (−34.00, −34.00)	−27.03 (−34.00, −12.86)	0.18
1 m	−15.43 (−29.92, −6.78)	−15.75 (−34.00, −5.52)	0.85
3 m	−9.12 (−13.69, −3.21)	−15.92 (−34.00, −4.48)	0.47
6 m	−10.96 (−14.35, −3.78)	−9.89 (−31.01, −5.99)	0.47
ΔMD (0–1 m)	16.68 (2.06, 23.08)	4.47 (0.00, 13.83)	0.10
ΔMD (0–3 m)	21.59 (12.61, 29.21)	2.25 (0.00, 11.39)	0.002^*^
ΔMD (0–6 m)	20.15 (13.09, 31.85)	8.36 (0.00, 15.15)	0.02^*^
*P* value (onset vs.6 m)	0.003^*^	0.046^*^	–
**VEP-A**, **μv, median (IQR)**			
Onset	4.78 (3.22, 6.05)	4.24 (0.00, 6.05)	0.61
1 m	6.41 (5.74, 8.82)	4.69 (0.00, 8.20)	0.28
3 m	7.43 (5.97, 9.39)	4.49 (0.00, 8.50)	0.19
6 m	7.08 (6.04, 10.72)	7.78 (0.00, 10.10)	0.69
ΔVEP-A (0–1 m)	3.22 (0.69, 8.65)	0.00 (−4.27, 4.03)	0.12
ΔVEP-A (0–3 m)	5.22 (2.76, 9.18)	0.00 (−4.27, 4.26)	0.029^*^
ΔVEP-A (0–6 m)	5.77 (3.39, 6.58)	0.00 (−4.27, 5.86)	0.23
*P* value (onset vs. 6 m)	0.036^*^	0.60	–
**Abnormal VEP-T**, ***n*** **(%)**			
Onset	6/8 (75.00%)	4/7 (57.14%)	0.61
1 m	5/8 (62.50%)	6/7 (85.71%)	0.57
3 m	4/8 (50.00%)	5/7 (71.43%)	0.61
6 m	6/8 (75.00%)	6/7 (85.71%)	>0.99
*P* value (onset vs. 6 m)	>0.99	0.56	–
**RNFL**, **μm, median (IQR)**			
Onset	108.50 (71.75, 120.00)	105.50 (74.25, 136.75)	0.91
1 m	83.50 (61.50, 110.50)	88.00 (47.75, 90.00)	0.62
3 m	60.00 (49.00, 77.50)	66.00 (42.50, 70.75)	1.00
6 m	51.50 (42.50, 73.75)	49.50 (39.00, 60.00)	0.57
ΔRNFL (0–1 m)	−11.00 (−50.25, −1.50)	−29.00 (−63.00, −9.00)	0.34
ΔRNFL (0–3 m)	−40.00 (−59.00, −18.25)	−55.00 (−74.00, −16.00)	0.52
ΔRNFL (0–6 m)	−51.50 (−67.25, −19.00)	−58.00 (−91.25, −19.50)	0.52
*P* value (onset vs. 6 m)	0.002^*^	0.012^*^	–

CP, intravenous cyclophosphamide; MP, intravenous methylprednisolone; BCVA, best-corrected visual acuity; MD, mean deviation; VEP-A, visual evoked potential amplitude; VEP-T, visual evoked potential latency; RNFL, retinal nerve fiber layer; IQR, interquartile ranges;

* P < 0.05.

Regarding the baseline clinical characteristics, no significant difference could be found between the two groups for any item (*P* > 0.05). Additionally, no significant difference could be found between the two groups regarding the total CP dosage in total and the initial MP intervention time point.

CP ≤ 30 days group showed significant improvement in BCVA as early as 1 m after onset; a significant difference were found between 1 m and baseline (*P* = 0.003). No significant difference from baseline was found after 1 m, which might indicate that the BCVA improved rapidly after remedial CP ≤ 30 days treatment and then level off. However, in the CP > 30 days group, no significant improvement to baseline could be found; no significant difference could be found at any follow-up point compared to baseline. This difference in BCVA improvement between the two groups was more obvious if the BCVA change to baseline was compared. The CP ≤ 30 days group showed significantly better ΔBCVA improvement at each follow-up time point compared to the CP > 30 days group (*P* Δ1 m = 0.02, *P* Δ3 m = 0.01, *P* Δ6 m = 0.02).

Within the CP ≤ 30 days group, significant improvement in MD was observed as early as 1 m after onset. Significant differences were found between 1 m and baseline (*P* = 0.008), 3 and 1 m (*P* = 0.033), and no significant differences were found between 6 and 3 m (*P* = 0.878). The CP > 30 days group also demonstrated significant improvement at 3 m compared to baseline (*P* = 0.043), but no significant difference was found afterward. Comparison between two groups: At every follow-up point, no statistically significant differences in either group were observed at any follow-up timepoint (1 m *P* = 0.85, 3 m *P* = 0.47, 6 m *P* = 0.47). Regarding MD change to baseline, significantly better improvement was found at 3 and 6 m in the CP ≤ 30 days group compared to the CP > 30 days group (*P* Δ1 m = 0.10, *P* Δ3 m = 0.002, *P* Δ6 m = 0.02).

Regarding VEP, VEP data of 15 patients were available for analysis: 8 patients in the CP ≤ 30 days group and 7 patients in the CP > 30 days group. Regarding VEP-A, significant improvement was found at 6 m compared to baseline in the CP ≤ 30 days group (*P* = 0.036), while no significant improvement was found in the CP > 30 days group. No significant difference was found between the two groups at any timepoint (*P* > 0.05). Regarding the VEP-A change to baseline, significantly better improvement was found at 3 m in the CP ≤ 30 days group compared to the CP > 30 days group (*P* Δ1 m = 0.12, *P* Δ3 m = 0.029, *P* Δ6 m = 0.23). Regarding the proportion of abnormal VEP-T, no significant difference was found between the two groups at any time point (*P* > 0.05).

Within the CP ≤ 30 days group, significant thinning in RNFL was found as early as 1 m after onset and continued to 6 m after onset: significant differences were found between 1 m and baseline (*P* = 0.005), 3 and 1 m (*P* = 0.005), and 3 and 6 m (*P* = 0.005). Within the CP > 30 days group, similar to the CP ≤ 30 days group, significant thinning was found as early as 1 m after onset and continued to 6 m after onset: significant differences were found between 1 m and baseline (*P* = 0.018), 3 and 1 m (*P* = 0.012), 3 and 6 m (*P* = 0.018). However, no significant difference could be found between the two groups at any follow-up point. Regarding RNFL change to baseline, no significantly better improvement was found in CP ≤ 30 days compared to CP > 30 days (*P* Δ1 m = 0.34, *P* Δ3 m = 0.52, *P* Δ6 m = 0.52).

#### Safety (Table 4)

Among patients who received CP, no serious adverse events were observed, and no patients terminated therapy for any adverse event. During CP treatment, nausea and fatigue (30%) occurred in 6 patients, but the symptoms were well tolerated and disappeared after 2–3 days, and no further medication was needed. Alopecia occurred in 2 patients (10%). Neutrophil, lymphocyte and platelet counts were slightly reduced but did not reach a clinically significant level, and no leukopenia (white blood cell count ≤ 3,500 cells/μL) was observed during the course of follow-up. Temporarily elevated alanine transaminase (ALT) levels occurred in 1 (5%) patient but returned to normal levels soon after treatment ended. No bladder bleeding, severe infection, malignancy or premature ovarian failure was observed.

**Table 4:** CP adverse events.

**Adverse events**	**Patients, *n* (%)**
Nausea	6 (30%)
Fatigue	6 (30%)
Alopecia	2 (10%)
Leukopenia	0
Elevated alanine transaminase (ALT)	1 (5%)
Bladder bleeding	0
Severe infection	0
Malignancy	0
Premature ovarian failure	0

CP, intravenous cyclophosphamide.

## Discussion

This is the first case-control study about the remedial efficacy of cyclophosphamide in the treatment of acute NMOSD-ON who had no response to MP treatment. Not only BCVA but also the VF, VEP, and RNFL were all analyzed to thoroughly evaluate different aspects of the efficacy of CP. The follow-up period was as long as 6 m after onset. The optimal treatment time window of CP was also analyzed and discussed.

### Advantage

NMOSD-ON is the first symptom of 35.3–58.1% (12, 13) of NMOSD patients, and it can cause irreversible damage to visual function; therefore, it is necessary to control the disease as soon as possible and reduce the loss of optic nerve fibers. MP is the first-line treatment of NMOSD-ON at the acute stage, due to broad-spectrum anti-inflammatory and immunosuppressive effects. However, at least 20% of patients with NMO do not respond to MP, especially in relapse cases. For these patients who were refractory to MP, other measures were also available, including IVIg, PE, rituxibmab, and eculizumab. Compared with other immunosuppressants which might take months to take effect, CP takes effect quickly (it will take effect in 1–3 m). Compared to PE/AI, CP inhibited cell proliferation and had a more lasting effect. Compared to rituximab, CP was also cheap, easy to obtain, safer and easy to monitor. Rituximab (RTX) is the most widely used drug for NMO relapse prevention nowadays. It takes RTX 2 weeks to deplete immature and memory B cells and show effects later on. As to CP, the number of white cells decreased to the lowest as early as 7 days after treatment. So, compared to RTX, CP is faster to display effect and might be more suitable for control of inflammation in acute phase. In addition, cyclophosphamide was more cost effective than RTX in China. As to Eculizumab, it is very expensive and unavailable to most patients. For IVIg, the effectiveness has been controversial.

### Effectiveness

The reason might be that glucocorticoids are not effective enough to suppress B cells that produce AQP4 autoantibodies (14). While CP has the advantage of not only playing a cytotoxic role in inhibiting cell DNA synthesis and cell division to kill lymphocytes at any stage of the cell division cycle, but also playing an immunosuppressive role in cellular immunity and humoral immunity by inhibiting the activity of lymphocytes. CP has a stronger inhibitory effect on B cells than T cells (15, 16) and could inhibit the proliferation and division of immune cells more strongly than MP.

### CP With SLE/SS

A single small case series of 10 patients with SLE- ON treated with CP in the acute phase found that 80% of the patients' visual acuity and visual field were improved (17). Case reports (6, 7) about CP treatment at acute phase of SLE/SS-related NMOSD showed successful experience in achieving disease stabilization and recovery. The EFNS in 2010 recommends the administration of CP as a second-line therapy for patients with NMO, especially in cases of association with SLE/SS (5). However, among the CP group in this study, none met the criteria of SLE, SS or any other immune disease. This indicated that CP's beneficial effect might not be restricted to SLE-associated or other immune disease-associated cases.

### Other Studies

There have been several prior studies of the effect of CP on NMOSD-LETM. The results were contradictory, which might be due to the considerable diversity of CP regimens, dose timing and patient selection bias. Some showed beneficial effects. In a series of 4 patients with AQP4 + NMOSD-LETM, significant EDSS improvement was achieved (6). Interestingly, an NMOSD-LETM case was once reported (7) in which significant recovery was achieved following a pulse of CP after the patient failed to respond to high-dose corticosteroids, PE, IVIg, and rituximab. In 2007, Greenberg et al. (18) found that 13 patients with transverse myelitis had significant EDSS improvement after CP+MP treatment. Among them, 84.6% were recurrent, and 69.2% were complicated with autoimmune disease. In another small case series (19) of 7 patients with NMOSD, pulse CP [1 g every 2 months (500–700 mg/m^2^), 2–7 times] + MP resulted in stabilization in only one patient.

### Remedial Window

Regarding the best time window for remedial treatment by adding CP, to the best of our knowledge, no such study on NMOSD-ON has ever been conducted. Adding CP ≤ 30 days after onset in this study showed a significantly better and faster improvement of BCVA and MD of VF and VEP-A than adding CP for more than 30 days (*P* < 0.05). This suggested that CP ≤ 30 days enhanced visual functional recovery in NMOSD-ON. A similar phenomenon was observed in a study of SLE-ON: CP was used 2–48 m after initial MP therapy, and still 80% (8/10) of the patients showed improvement (17). This might indicate that the remedial time window for CP might be quite early. The mechanism might be B cell early suppression by CP. However, in those patients who received CP > 30 days, beneficial effects were also found, which might be due to the surviving optic nerve fibers that were rescued by delayed CP treatment. When the anti-inflammatory effect of MP was not strong enough and decreased with time, inflammatory factors production and inflammatory cells proliferation might continue, especially the B cells that produce AQP4 antibodies, which might survive for weeks to months, and continue to inhibit B cells, producing beneficial effects.

### Safety

The CP regimen recommended by the EFNS is 7–25 mg/kg iv every month (350–1,250 mg/m)over a period of 6 m (5). In this study, the CP dosage was approximately 375 mg/mm^2^ (0.6 g), taken over a shorter period (1–4 w) than recommended by the EFNS. This is the widely accepted CP regimen in general autoimmune diseases: weekly use [after the administration of CP, lymphocytes and granulocytes usually reached the lowest point on the 7th day and 14th day, respectively (20)], 375 mg/mm^2^ (0.6 g) each time (according to the patient's body weight 50–60 kg, the average body surface area 1.6 m^2^ is calculated by 375 mg/mm^2^), the total course of treatment is 4 weeks, and the number of doses is 1–4 (according to the improvement of the disease and the stability of the patient's condition).The European Euro-Lupus low-dose CP regimen (15) (0.5 g, once every 2 weeks, 6 consecutive times, cumulative dose CP of 3 g) is sufficient to temporarily inhibit the proliferation of B immune cells. However, this study showed that 15 of 20 patients were treated with CP (cumulative dose of CP < 3 g) gained a better improvement in visual functional index. All the patients included in this study were Han Chinese. This may be the reason why a lower dose can still obtain a good effect. For doses higher than 0.6 g, such as 0.8–1.0 g (500–625 mg/mm^2^) each time, whether it has a better effect needs to be further verified.

CP has a low selectivity to normal cells, which causes it to produce unavoidable adverse reactions; the incidence of these reactions is related to the cumulative dose of CP, medication time and age (21). The safety issue of CP is a major concern, including serious adverse events such as bone marrow suppression, bladder toxicity, severe infection, malignancy and premature ovarian failure. In this study, no serious adverse reactions were observed. The reason might be the very low total dosage used: CP 375 mg/mm^2^ (0.6 g) for just 1–4 weeks, which made the cumulative dose no more than 3 g. In 2016, Yan Xu et al. (22) gave CP 0.4 g/w to 41 patients with chronic NMOSD for 30 weeks, which means that the cumulative dose went as high as 12 g, and CP-related adverse reactions occurred in 14 cases (34.1%), including leukopenia, elevation of liver enzymes, amenorrhea, hemorrhagic cystitis in 1 case (2.4%), gastrointestinal disorder in 1 case (2.4%), and thrombocytopenia in 1 case (2.4%). The gonadal injury was not tested in this study because it had been proven that a cumulative adult CP dose of 6 g or less is relatively safe and does not affect the gonadal reserve (23). However, the possibility of gonadal injury was discussed with patients before use.

This study has the following limitations: (1) As this was a single-center, retrospective case study, it was difficult to reach a definitive conclusion. Furthermore, a prospective multicenter random clinical trial of CP in NMOSD-ON is needed. (2) No comparison was made to other remedial therapies, such as IA, PE, or exhausted B cell therapy. (3) This study analyzed only adult NMOSD-ON, not pediatric NMOSD-ON. (4) Only AQP4-positive NMOSD-ON was included in this study, but the therapeutic response of AQP4-negative NMOSD-ON was not analyzed. (5) Details of accurate timing from symptom onset to acute CP treatment needed to be analyzed, such as CP ≤ 14 days and CP > 14 days.

### Conclusion

This study suggested that cyclophosphamide might be an effective remedial therapy when glucocorticoid pulse therapy alone is not effective in patients with acute NMOSD-ON attacks. Cyclophosphamide may be more effective 30 days after onset.

## Data Availability Statement

The raw data supporting the conclusions of this article will be made available by the authors, without undue reservation.

## Ethics Statement

The studies involving human participants were reviewed and approved by Ethics Committee of Zhongshan Ophthalmology Center of Sun Yat-sen University. The patients/participants provided their written informed consent to participate in this study.

## Author Contributions

HY and LW designed and performed the study. LW, KL, XT, LZ, YZ, XL, and YF were responsible for data collection. LW analyzed the data. HY, WQ, and LW drafted the manuscript. All authors read, critically revised, and approved the final manuscript.

## Conflict of Interest

The authors declare that the research was conducted in the absence of any commercial or financial relationships that could be construed as a potential conflict of interest.
